# The complexity of safety: Embracing systems engineering in radiation oncology

**DOI:** 10.1002/acm2.14622

**Published:** 2025-02-07

**Authors:** Lawrence M. Wong, Todd Pawlicki

**Affiliations:** ^1^ Department of Radiation Medicine & Applied Sciences University of California San Diego La Jolla California USA

In the past 30 years, radiation oncology has posted remarkable milestones such as the introduction of intensity modulated treatments, image‐ and surface‐guided localization, FLASH, and online plan adaptation based on the anatomy of the day. Indeed, the complexity of clinical practice is increasing exponentially, and with increasing complexity comes increasing risk. How do we best grapple with these changes while continuing to focus on patient safety improvement in radiation oncology?

AAPM TG‐100 was published about 8 years ago to help make radiation oncology safer and more efficient.^1^ Despite those laudable intentions, AAPM TG‐100′s emphasis on risk‐based analysis techniques is not sufficient to address the impact of complexity on safety in radiation oncology. The fact that many errors are caused by workflow and process deviations, rather than device or software failures, highlights the limitations of an approach that has a large focus on estimating failure probabilities. The complexity of radiation oncology, which includes the variability of human behavior, requires a more nuanced and multifaceted approach to ensuring patient safety. Systems engineering (or systems thinking) can help answer this and many other questions critical to understanding of how to deal with the complex system that is radiation oncology.

Recognizing that a system is complex is an important first step in expanding our safety toolbox beyond probability‐based risk analysis techniques to understanding safety. Complex systems involve a high degree of interconnectedness, interdependence, and non‐linearity among system components.^2^, ^3^ What occurs in one step of the radiation oncology process of care can have disastrous consequences in another step even though neither step has failed. For example, consider a prescription for palliative treatment to the spine (T11 – L1) using 6 MV and of total dose of 3000 cGy at 300 cG/fx to the isocenter using an isocentric setup and the beam was planned (and delivered) to enter the patient from the anterior with the isocenter located in the vertebral body. In this example, no step in the process has explicitly failed but this is clearly an error. It is the interconnectedness and interdependence of the steps that contributed to the error.

In complex systems, surprising phenomena can arise that cannot be predicted by examining individual parts in isolation, through a concept known as emergence. Emergence is a foundational concept of systems thinking. It is the idea that some system properties only exist when the whole system is considered but not at the level of its individual components.^3^ Emergent properties come about through the interactions between system components. An example is that treatment effectiveness can only be assessed when the entire radiation oncology system is considered but not at the level of the subsystems such as treatment planning or treatment delivery. Treatment effectiveness suffers even when the most accurate dose calculation algorithm is applied but the patient setup is suboptimal and delivers excess dose to normal tissues. Patient safety is also an emergent property of the complex system of radiation oncology.

When we talk about addressing patient safety in radiation oncology, we must appreciate the difference between a complicated system and a complex system.^4^ The safety solution space in a complicated system is stationary or fixed. If you fully understand the individual components, then you can create a safe system. Think of a linear accelerator—extremely complicated—but the relationships between components are fixed and the structure of the system can be fully determined. After all the effort over the years, linear accelerators hardly ever fail catastrophically. In a complex system, the solution space is constantly changing. This is why workflows and processes are the biggest safety challenges in radiation oncology—the possible solution space is constantly being changed, in part, by some of the system components themselves (e.g., humans). This is one of the reasons why a probabilistic‐based risk assessment approach to prioritizing safety interventions, such as failure modes and effects analysis (FMEA), will always have limited reliability and effectiveness. Probabilities of detection and occurrence cannot be estimated with any long‐term accuracy because they depend on local conditions that change over time, sometimes even from one moment to the next. The FMEA procedure also lacks a systematic methodology for analyzing component interactions and identifying hazards that may lead to accidents as it does not provide a comprehensive understanding of how interconnected components need to operate in order to achieve the goals of the system. Ultimately, the whole FMEA procedure rests on an accident model that is known to have limited effectiveness when applied to complex systems.

Systems engineering is an approach to understanding and dealing with complex systems. It emphasizes analyzing the interconnectedness of various components, relationships, and system dynamics.^3^, ^5^, ^6^ Furthermore, systems can only be fully understood by considering all aspects together, including both technical and non‐technical (such as social) factors, in their entirety. Because the system must be considered as a whole and emergent properties can arise from the interactions of system components, systems engineering recognizes that closed‐loop control (discussed in more detail below) is essential for establishing and maintaining operational stability.

Closed‐loop control seeks to address the fact that accidents often result from unsafe interactions between system components, which can include equipment, software, people, or specific steps within a process.^5^, ^6^ To maintain a system in a safe state, a control system with a diverse set of control actions is necessary to respond to various changes within the system. In this paradigm, control can exist on a spectrum, and the most effective approach may be a subtle influence, such as a ‘soft’ nudge, particularly in social contexts. A closed‐loop control system operates in a continuous feedback loop, where the output is constantly measured and adjusted to align with a predetermined target value or intended outcome. This system comprises three essential elements: a sensor to detect the output, a controller to evaluate the output against the desired reference value and determine the necessary corrective action, and an actuator to modify the system input based on the controller's instructions. The process repeats continuously. Closed‐loop control systems are widely used in various fields, including process control, robotics, and automation, and offer several benefits, such as improved accuracy, increased stability, and optimized performance. By continuously monitoring and adjusting the output, these systems can ensure that the desired outcome is achieved and maintained.

When complexity is not well‐managed, a system becomes more susceptible to producing unintended outcomes, that is, accidents. One area of increasing complexity in radiation oncology is artificial intelligence and automation. The integration of artificial intelligence and automation alters the control dynamics between humans and equipment, sometimes reducing human control authority. While automation can significantly improve safety, it also creates new challenges.^5^ For instance, human operators may only be granted control authority when a situation exceeds the capabilities of automation, leaving them unprepared to prevent accidents due to limited control, awareness, or reaction time. In such cases, attributing the accident to human error oversimplifies the issue and fails to address the underlying complexities. Instead, we need to investigate the safety implications of human oversight in automated systems more thoroughly using a systems engineering approach. The responsibilities of human operators have actually increased with automation, as they must now oversee multiple processes simultaneously, balance competing goals, and perform tasks under conditions not covered by standard operating procedures. In fact, clinicians may need to deviate from procedures or implement workarounds to prevent accidents. Simply identifying procedural violations as the cause of accidents neglects the interconnectedness and dynamic factors that are at play. While we can learn from industries like aviation, which have extensive experience with automation, the unique characteristics of radiation oncology (and healthcare in general) require more research on patient safety in complex systems.

Since accidents can occur differently in complex systems compared to complicated systems, new models of accident causation and analysis techniques have been developed by safety researchers. A comprehensive review of these, alongside traditional models and techniques, can be found in the accompanying review article in this issue of the *Journal of Applied Clinical Medical Physics*.^7^ The advances in safety engineering arises from viewing accidents with a systems engineering lens and incorporating many of the systems concepts introduced above. By investigating and adopting safety improvement approach based in systems engineering, we can gain a deeper understanding of radiation oncology and make more informed decisions that positively impact patient safety as the complexity of clinical practice continues its exponential increase in the coming years.

## AUTHOR CONTRIBUTIONS

Lawrence Wong and Todd Pawlicki jointly performed conceptualization, investigation, writing, and visualization of the work and agree to be accountable for all aspects of the work.

## CONFLICT OF INTEREST STATEMENT

Lawrence Wong and Todd Pawlicki receive research funding from Varian Medical Systems. Todd Pawlicki has received speaking honoraria from Varian Medical Systems and is a founding partner of Image Owl, LLC.
